# Use of prescription medicines in Australian women of child-bearing age

**DOI:** 10.1186/s40360-015-0033-x

**Published:** 2015-12-07

**Authors:** Svetla Gadzhanova, Elizabeth Roughead

**Affiliations:** Quality Use of Medicines and Pharmacy Research Centre, Sansom Institute, School of Pharmacy and Medical Sciences, University of South Australia, Adelaide, Australia

**Keywords:** Medicines, Women of child-bearing age, Australia

## Abstract

**Background:**

This study aimed to examine current utilisation of prescribed medicines amongst Australian women of child-bearing age, with a particular focus on the extent of use of medicines in Category D and X risk groups, which are moderate and high risk teratogens, respectively. The use of those medicines may pose risk of birth defects in pregnant women.

**Methods:**

A retrospective cross-sectional study was undertaken involving all women of child-bearing age (15 to 44 years) who were dispensed medicines in 2013 using the 10 % random sample of dispensing data from the Australian Government Department of Human Services. Dispensing patterns were reported by medicine, therapeutic class, pregnancy risk category and women’s age.

**Results:**

Over one-third of women aged 15 to 44 years received at least one prescribed medicine in 2013. Psychoanaleptics, antibiotics and analgesics were the top three classes. Around 9 % of all dispensings were for medicines from risk category D, with statins, agents acting on renin-angiotensin system, and some anti-epileptic agents being the most commonly used. Both statins and agents acting on renin-angiotensin system showed increasing use with age, estimated to be 35,600 women nationally for each group. Collectively between 2 % and 4 % of women used anti-epileptics from risk category D in each year of age, with overall use estimated to be 51,000 women nationally. Below 1 % of all dispensings were for category X medicines, mainly isotretinoin.

**Conclusions:**

It is important for medical practitioners to offer counselling around pregnancy planning and the risk of birth defects when prescribing moderate or high risk teratogens to women in child-bearing age. For the antihypertensives and some anti-epileptics, alternative medicines with lower risk categorization are available.

## Background

Six percent of women of child-bearing age (15 to 44 years) gave birth in Australia in 2012 [1]. Pregnancy creates challenges for peri-conception care as there is potential for unintentional exposure to teratogenic substances, including medicines. It is important for prescribers to be aware of teratogenic drug-induced effects in women who are or could become pregnant. Prescribers are likely to be aware of the very few medicines that are considered “high-risk” teratogens and should be absolutely avoided in pregnancy (e.g. isotretinoin), which cause major birth deformities at rates of 1 in 4 exposed foetuses [2]. The extent of use of medicines that are considered lower or moderate risk of harm in women of child-bearing age is less well studied. The “moderate-risk teratogens” cause birth defects in a smaller proportion of pregnancies but still have a 5 to 20 fold increase in specific risk [2] (e.g. carbamazepine and sodium valproate) and in many cases safer alternatives are available.

Category D medicines are those which may be considered moderate risk teratogens and are defined as medicines which have caused, or are suspected to have caused an increased incidence of human foetal malformations or irreversible damage [3]. Some commonly prescribed medicines, including statins and the antihypertensive medicines acting on the renin-angiotensin system are category D medicines. In the antidepressant class, paroxetine is considered category D, whilst amongst antiepileptics, topiramate, lamotrigine, carbamazepine, sodium valproate, phenytoin and oxcarbazepine are all category D. For many of these medicines, alternative agents are available and should be considered where women are at risk of unplanned pregnancies.

A systematic review of Australian studies using national health data identified that use of medicines in pregnant women is an area where more research is required [4]. The majority of prior Australian studies from Western Australia (WA) linked pregnancy events in WA from 2002 to 2005 to national pharmaceutical claims (Pharmaceutical Benefits Scheme (PBS) data). One of the studies reported that in 28 % of all pregnancy events, women were exposed to a PBS medicine while pregnant [5]. While studies have examined medicine use in women during pregnancy, we located no Australian studies that have examined medicine utilisation for women of child-bearing age, particularly the use of medicines that may be teratogenic.

## Aim of the study

This study aimed to examine current utilisation of prescribed medicines amongst Australian women of child-bearing age, with a particular focus on the extent of use of medicines in category D and X risk groups, which are moderate and high risk teratogens, respectively.

## Method

De-identified national pharmacy claims data from the Australian Government Department of Human Services were utilised, providing a 10 % random sample of people who had medicines subsidised and dispensed under the Pharmaceutical Benefits Scheme (PBS) [6]. Under the PBS, the Australian Government subsidised the medicine cost above the co-payment threshold. The co-payment is the amount paid by the patients towards the cost of their PBS medicines. From 1 January 2012, general patients pay up to $35.40 for most PBS medicines or $5.80 if they have a concession card. PBS data is collected from pharmacies and public and private hospitals. It includes information on patient age, gender, beneficiary status (general or concessional beneficiary status), as well as dispensing information, which includes date of supply, drug code, therapeutic class, generic name, form, quantity dispensed and number of repeats. Since 2012, PBS data represents full capture of dispensing records for both general and concessional beneficiaries. Medicines were coded in the dataset according to the World Health Organization Anatomical Therapeutic Chemical (ATC) classification system [7].

A retrospective cross-sectional study was undertaken involving all women of child-bearing age (15 to 44 years) who were dispensed medicines in 2013. Both general and concessional beneficiaries were included as data comprised all subsidized medicines, including those below the co-payment threshold. The pregnancy risk category for each medicine was determined using the pregnancy database provided by the Therapeutic Goods Administration, Australian Government Department of Health [3]. There are 7 categories under the Australian categorisation of the risk of drug use in pregnancy (Table 1) [3]. Drugs from category A are considered to be safe for use; drugs from category B1, B2, B3 have not been shown to increase the frequency of malformation or harmful effects on the human foetus; drugs from category C may cause harmful effects on the human foetus without causing malformations. Drugs from categories D and X are considered moderate to high risk medicines as they increase the incidence of human foetal malformations and lead to irreversible and permanent damage. For combination products the categorisation was based on the component with the most restrictive category. For this analysis, we excluded medicines where there were less than 400 dispensings per year, which accounted for less than 0.03 % of all dispensings for each medicine and represented 2.8 % of total use.

**Table 1:** Definitions of the Australian categories for prescribing medicines in pregnancy [3]

Category A
Drugs which have been taken by a large number of pregnant women and women of childbearing age without any proven increase in the frequency of malformations or other direct or indirect harmful effects on the fetus having been observed.
Category B1
Drugs which have been taken by only a limited number of pregnant women and women of childbearing age, without an increase in the frequency of malformation or other direct or indirect harmful effects on the human fetus having been observed.
Studies in animals have not shown evidence of an increased occurrence of fetal damage.
Category B2
Drugs which have been taken by only a limited number of pregnant women and women of childbearing age, without an increase in the frequency of malformation or other direct or indirect harmful effects on the human fetus having been observed.
Studies in animals are inadequate or may be lacking, but available data show no evidence of an increased occurrence of fetal damage.
Category B3
Drugs which have been taken by only a limited number of pregnant women and women of childbearing age, without an increase in the frequency of malformation or other direct or indirect harmful effects on the human fetus having been observed.
Studies in animals have shown evidence of an increased occurrence of fetal damage, the significance of which is considered uncertain in humans.
Category C
Drugs which, owing to their pharmacological effects, have caused or may be suspected of causing, harmful effects on the human fetus or neonate without causing malformations. These effects may be reversible. Accompanying texts should be consulted for further details.
Category D
Drugs which have caused, are suspected to have caused or may be expected to cause, an increased incidence of human fetal malformations or irreversible damage. These drugs may also have adverse pharmacological effects. Accompanying texts should be consulted for further details.
Category X
Drugs which have such a high risk of causing permanent damage to the fetus that they should not be used in pregnancy or when there is a possibility of pregnancy.

We specifically examined medicines with potential teratogenic effect. Analysis included the antidepressants: paroxetine (category D), fluoxetine and sertraline (both category C). A recent meta-analysis found paroxetine and fluoxetine were associated with excess risk of birth defects, but previously reported birth defects were not confirmed for sertraline [8]. With regards to anti-epileptics, topiramate, lamotrigine, carbamazepine, sodium valproate, phenytoin and oxcarbazepine are all category D. Agents acting on renin-angiotensin system are all category D, with alternative antihypertensives available. Statins are also category D, however, limited pharmacological alternatives are available, with non-pharmacological therapies an alternative option for some women.

Prescribing patterns were reported by medicine therapeutic class [7], pregnancy risk category [3] and patient year of age. To extrapolate to national estimates of prevalence of use, we multiplied the results by a factor of ten, because our sample was 10 %, and used the Australian Bureau of Statistics population estimates of women age 15 to 44 years at 30 June 2013 [9]. Birth rates were also presented based on number of births by mother’s age in 2013 [10]. The use of selected medicines was presented as the rate of unique (distinct) women who received that medicine in 2013 as a proportion of all unique women in the given age group with at least one PBS subsidised medicine in 2013. Student’s *t*-test was used to compare means between any two groups.

## Ethics

The study was approved by the Department of Human Services (DHS) External Request Evaluation Committee for analysis of PBS data. All analysis were conducted using de-identified data and in accordance with DHS Privacy Policy.

## Results

**Fig. 1:**
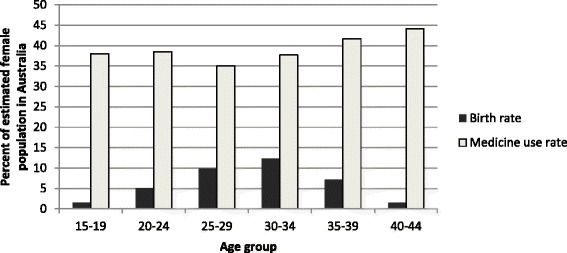
Birth rate and rate of unique women dispensed at least one PBS subsidised medicine in 2013 as proportion of estimated female resident population at 30 June 2013 (by age)

There were 1,282,720 dispensings for 188,412 unique women aged 15 to44 years in 2013 (mean age 30.3, SD = 8.6). This approximates to 39 % of women of child-bearing age nationally receiving at least one dispensed medicine. Figure 1 presents the estimated birth rates and rates of women dispensed at least one medicine by age groups. One-third or more of the women in each age group received at least one medicine in 2013, however those with the highest birth rates (25 to 34 years) had slightly lower medicine use.

We identified and categorized 265 medicines which had 400 or more dispensings. Table 2 presents the proportion of women and frequency of use of medicines by pregnancy risk category. Medicines from categories A, B3 and C were the most commonly dispensed, with proportions ranging from 21 % (category A) to 27 % (category C). 10 % of all dispensings were for medicines from category D (9.3 %) or X (0.6 %) with women receiving category X medicines (predominantly isotretinoin) being younger.

**Table 2:** PBS medicines dispensed in women aged 15 to 44 in 2013 by pregnancy risk category

Pregnancy risk category	Dispensings (Total *N* = 1282720)	Unique women (% of the total *N* = 188412)	Mean age (years)
A	270169 (21.1 %)	48.6 %	29.9
B1	95685 (7.5 %)	23.0 %	30.6
B2	110093 (8.6 %)	11.9 %	31.1
B3	298430 (23.3 %)	52.8 %	30.4
C	343917 (26.8 %)	28.9 %	32.0
D	118943 (9.3 %)	15.0 %	31.3
X	7901 (0.6 %)	1.2 %	24.9*

The percentages of dispenses sum up to 97.2 % as we categorized only medicines with 400 or more dispensings in 2013

The percentages of unique women sum up to over 100 as many women had medicines from multiple categories

*Significantly younger than women dispensed medicines from any one of the remaining risk categories (student *T*-test, *p* < 0.0001)

**Fig. 2:**
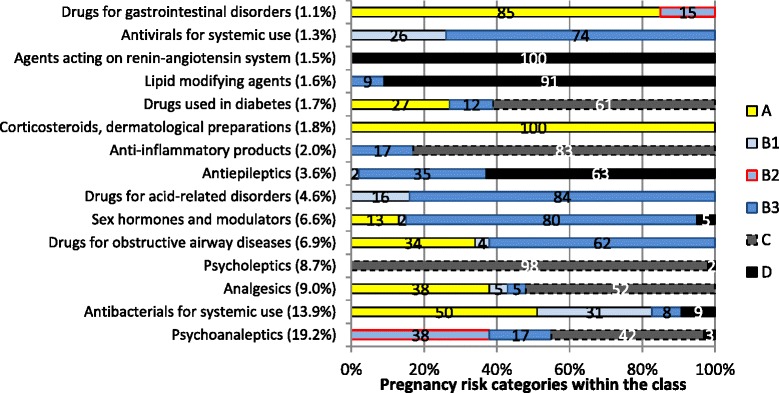
Top 15 therapeutic classes (by ATC category, level2), ordered by percentage of all dispensings in 2013; presented by pregnancy risk category (visualize pregnancy category proportions > =2 % within a class)

The top 15 therapeutic classes dispensed to women aged 15 to 44 years, contributed to 84 % of all dispensings in the PBS sample (Fig. 2). One fifth of all dispensings were for psychoanaleptics, followed by antibiotics (14 %), analgesics (9 %) and psycholeptics (9 %). We examined the medicines within each of those 15 therapeutic groups by risk category and generic name (Fig. 2 and Table 3). For the first group of psychoanaleptics, most dispensings were for antidepressants from C category, followed by B2 category (Fig. 2). Table 3 lists the individual medicines and shows that desvenlafaxine was most common, followed by sertraline.

**Table 3:** Top 15 therapeutic classes (by ATC category, level2) by pregnancy risk category and generic name ordered by descending number of dispensings (ND)

Therapeutic group	Category A	Category B1	Category B2	Category B3	Category C	Category D
Psycho-analeptics (NW : 42095) (ND : 245976)		Reboxetine (0.6 %)	Desvenlafaxine (16.2 %)	Duloxetine (9.5 %)	Sertraline (14.2 %)	Paroxetine (3.1 %) (*N* = 6574 dispenses)
			Venlafaxine (13.3 %)	Mirtazapine (6.4 %)	Escitalopram (14.0 %)	
			Methylphenidate (1.3 %)	Dexamphetamine (1.1 %)	Fluoxetine (8.8 %)	
				Moclobemide (0.3 %)	Amitriptyline (7.0 %)	
					Citalopram (5.5 %)	
					Fluvoxamine (2.0 %)	
					Dothiepin (0.7 %)	
					Doxepin (0.4 %)	
					Clomipramine (0.3 %)	
					Nortriptyline (0.3 %)	
					Imipramine (0.2 %)	
Anti-bacterials for systemic use (NW: 106522) (ND: 178481)	Amoxycillin (26.0 %)	Amoxycillin with Clavulanic acid (17.0 %)	Dicloxacillin (1.5 %)	Trimethoprim (5.5 %)	Trimethoprim with sulphamethoxazole (1.4 %)	Doxycycline (6.4 %) (*N* = 12651)
	Cefalexin (22.5 %)	Roxithromycin (8.4 %)		Clarithromycin (4.0 %)		Minocycline (1.3 %) (*N* = 3103)
	Phenoxymethylpenicillin (5.1 %)	Flucloxacillin (3.6 %)		Ciprofloxacin (0.3 %)		
	Erythromycin (4.2 %)	Cefaclor (2.5 %)		Norfloxacin (0.3 %)		
	Clindamycin (1.2 %)	Azithromycin (1.9 %)				
	Nitrofurantoin (0.5 %)	Cefuroxime (0.4 %)				
	Hexamine (0.3 %)					
Analgesics (NW: 31705) (ND: 115460)	Codeine with paracetamol (53.2 %)	Rizatriptan (2.3 %)		Sumatriptan (3.3 %)	Oxycodone (17.5 %)	
	Paracetamol (11.1 %)	Pizotifen (1.9 %)		Zolmitriptan (0.4 %)	Tramadol (13.5 %)	
		Eletriptan (1.1 %)		Naratriptan (0.3 %)	Oxycodone comb. (2.3 %)	
					Buprenorphine (1.7 %)	
					Fentanyl (1.1 %)	
					Morphine (0.8 %)	
					Hydromorphone (0.4 %)	
Psycholeptics (NW: 20157) (ND:111528)					Diazepam (37.2 %)	Lithium (3.0 %) (*N* = 1978)
					Quetiapine (19.8 %)	
					Temazepam (22.2 %)	
					Prochlorperazine (15.7 %)	
					Olanzapine (8.0 %)	
					Oxazepam (7.3 %)	
					Alprazolam (4.9 %)	
					Risperidone (3.6 %)	
					Aripiprazole (3.3 %)	
					Nitrazepam (2.1 %)	
					Paliperidone (1.5 %)	
					Chlorpromazine (1.1 %)	
					Asenapine (1.0 %)	
					Amisulpride (0.9 %)	
					Ziprasidone (0.9 %)	
					Pericyazine (0.9 %)	
					Clozapine (0.7 %)	
					Zuclopenthixol (0.5 %)	
Drugs for obstructive airway diseases (NW: 34757) (ND: 88275)	Salbutamol (30.9 %)	Tiotropium (1.1 %)		Fluticasone with salmeterol (33.9 %)		
	Budesonide (2.1 %)	Ipratropium (1.1 %)		Budesonide with eformoterol (26.0 %)		
	Terbutaline (1.4 %)	Nedocromil (1.1 %)		Fluticasone (4.2 %)		
				Ciclesonide (1.0 %)		
Sex hormones and modulators of the genital system (NW: 46830) (ND: 84543)	Medroxyprogesterone (10.9 %)^a^	Oestradiol (0.9 %) (estrogen)		Levonorgestrel with ethinyloestradiol (56.3 %)^a^		Norethisterone (3.4 %) (*N* = 3504)^b^
				Etonogestrel (19.3 %)^a^		Medroxyprogesterone (1.1 %) (*N* = 1001)^b^
				Norethisterone with ethinyloestradiol (5.5 %)^a^		
				Levonorgestrel (4.4 %)^a^		
				Norethisterone (1.1 %)^a^		
				Clomiphene (0.6 %) (ovulation stimulant)		
Drugs for acid-related disorders (NW: 19444) (ND: 59365)		Rabeprazole (9.8 %)		Esomeprazole (56.4 %)		
		Ranitidine (7.2 %)		Pantoprazole (16.4 %)		
				Esomeprazole and amoxicilan and clarithromycin (9.2 %)		
				Omeprazole (6.6 %)		
				Lansoprazole (1.2 %)		
				Nizatidine (0.7 %)		
Antiepileptics (NW: 7784) (ND: 46092)		Gabapentin (1.9 %)		Pregabalin (29.0 %)	Clonazepam (1.9 %)	Valproate (29.1 %) (*N* = 8364)
				Levetiracetam (7.8 %)		Topiramate (14.3 %) (*N* = 7607)
				Lacosamide (1.0 %)		Lamotrigine (11.6 %) (*N* = 8803
						Carbamazepine (10.5 %) (*N* = 3153)
						Phenytoin (1.3 %) (*N* = 472)
						Oxcarbazepine (0.7 %) (*N* = 410)
Anti-inflammatory products (NW: 14676) (ND: 25747)				Celecoxib (13.9 %)	Ibuprofen (23.7 %)	
					Diclofenac (21.1 %)	
					Meloxicam (20.6 %)	
					Naproxen (13.1 %)	
					Mefenamic acid (10.6 %)	
					Indomethacin (3.9 %)	
					Piroxicam (1.5 %)	
Corticosteroids, dermatological preparations (NW: 14759) (ND: 23389)	Mometasone (37.7 %)					
	Betamethasone (34.7 %)					
	Methylprednisolone (21.3 %)					
	Hydrocortisone (13.4 %)					
	Triamcinolone (8.4 %)					
Drugs used in diabetes (NW: 6792) (ND: 21668)	Insulin aspart (23.8 %)			Insulin glargine (16.0 %)	Metformin (40.4 %)	
	Insulin (human) (8.7 %)			Sitagliptin (1.8 %)	Gliclazide (5.5 %)	
	Insulin lispro (5.7 %)				Metformin with sitagliptin (3.0 %)	
	Insulin detemir (5.6 %)				Exenatide (1.9 %)	
	Insulin aspart/protamine (3.3 %)				Metformin with vildagliptin (1.4 %)	
Lipid modifying agents (NW: 3842) (ND: 20916)				Fenofibrate (5.6 %)		Rosuvastatin (45.3 %) (*N* = 9225)
				Ezetimibe (3.7 %)		Atorvastatin (39.3 %) (N-7760)
						Simvastatin (4.0 %) (*N* = 750)
						Simvastatin and ezetimibe (3.1 %) (*N* = 680)
						Atorvastatin and amlodipine (2.3 %) (*N* = 544)
Agents acting on renin-angiotensin system (NW: 3560) (ND: 19330)						Perindopril (29.7 %) (*N* = 5191)
						Candesartan (12.8 %) (*N* = 2154)
						Irbesartan (12.2 %) (*N* = 2349)
						Telmisartan (12.1 %) (*N* = 2183)
						Ramipril (10.6 %) (*N* = 2030)
						Perindopril and amlodipine (7.4 %) (*N* = 1412)
						Olmesartan (5.8 %) (*N* = 949)
						Irbesartan with hydrochlorothiazide (HCTZ) (5.1 %) (*N* = 866)
						Perindopril with indapamide (4.4 %) (*N* = 789)
						Telmisartan with HCTZ (4.2 %) (*N* = 722)
						Candesartan with HCTZ (4.2 %) (*N* = 685)
Antivirals for systemic use (NW: 6837) (ND: 16556)		Famciclovir (32.6 %)		Valaciclovir (60.2 %)		
				Aciclovir (8.1 %)		
Drugs for gastrointestinal disorders (NW: 8874) (ND: 13074)	Metoclopramide (88.6 %)		Domperidone (11.4 %)			

The % reported for each medicine is from the number of unique women (NW) receiving the given therapeutic group. If the percentages of unique women sum up to over 100 it denotes that some women received multiple medicines from the same therapeutic class in 2013. For medicines in pregnancy risk category D we also reported the number of dispenses

^a^hormonal contraceptives for systemic use (ATC G03A); ^b^ progestogens (ATC G03D)

### High and moderate risk teratogens - categories D (contraindicated) and X (absolutely contraindicated)

Category D medicines contributed to 9.3 % of all dispensings in 2013, while category X – to 0.6 %. There were 30,039 unique women (16 % of all 188,412 women) who had medicine(s) from category D or X at some point of time in 2013. Doxycycline was the most commonly used drug from category D (by volume of dispensings), followed by rosuvastatin, lamotrigine, sodium valproate, atorvastatin and topiramate. Isotretinoin (anti-acne preparation for systemic use) was the most commonly prescribed drug from category X (84 % of all dispensings in this category), followed by leflunomide (immunosuppressant).

**Fig. 3:**
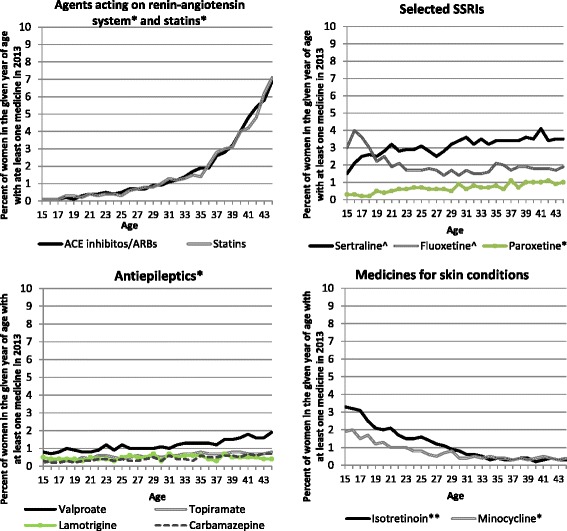
Use of selected medicines from category C, D and X which may pose increased risk of birth defects (by age). Legend: ^ Category C; * Category D; ** Category X; ACE inhibitors – Angiotensin converting enzyme inhibitors; ARBs – angiotensin II receptor antagonists. SSRIs – Selective serotonin reuptake inhibitors

The use of specific medicines that may pose increased risk of birth defects are presented in Fig. 3. There was increasing use of agents acting on renin-angiotensin system and statins with age, both showing use below 1 % in women under 30 years, 1 % to 4 % between 31 and 40 years, and up to 7 % in those aged 44 years. This equates to an estimated 35,600 women nationally for each of the two drug groups. Paroxetine, a Category D medicine, was used by 1 % or less of women in each year of age – this equates to 1,100 women nationally aged 15 to 20; 3,800 women aged 21 to 30, and 8,200 women aged 31 to 44. Fluoxetine had the highest rates of use in women below 20 years, and sertraline was used by 4 % or less of women in each year of age. Rates of use of the anti-epileptics (category D) were similar across all age groups and agents, with collective use between 2 % and 4 % in each year of age. Their overall use was estimated to be 51,000 women nationally, with valproate being used by 22,700 women, topiramate by 11,100, lamotrigine by 9,000, and carbamazepine by 8,200 women. Use of isotretinoin (category X) and minocycline (category D) were highest in younger women, used by an estimated 20,700 women nationally for isotretinoin and 13,600 women for minocycline.

## Discussion

We found that over one-third of the women aged 15 to 44 years in Australia had received at least one medicine in 2013. Those aged 25 to 34 years had the highest birth rates and slightly lower medicine use.

We found that the majority of use was for medicines considered relatively safe in pregnancy (categorised in A or B risk groups). Our results have some similarities to previous Australian research assessing medicine use in pregnant women. The rates for psychoanaleptics, antibiotics, analgesics and psycholeptics that we found among women of child-bearing age were similar to that found previously in pregnant women [5]. In both studies similar proportions were for Category C medicines (27 % in our study compared with 26 % as reported by [5]). Higher rates of Category A medicines were found among pregnant women (43 %) [5] compared to 21 % in our study. The use of iron with folic acid (anti-anemic preparation) and metoclopramide (for nausea and vomiting) in pregnancy are likely to account for this difference. Category D medicines contributed to 9 % of all dispensings in our study compared to 5 % of all dispensings in pregnant women [5]. Both studies found very low use of category X medicines (below 1 %). The relatively low use of category D and X medicines in pregnant women and in all women of child-bearing age implies caution when prescribing those moderate or high risk teratogens to women.

Women of child-bearing age may have chronic condition (e.g. hypertension or epilepsy) which require medicine(s) therapy. Hypertensive disorders are common and management of chronic hypertension is essential. As some commonly prescribed antihypertensive drugs such as agents acting on on renin-angiotensin system are category D, they should be avoided before conception and during pregnancy [11]. Pre-pregnancy counselling is essential and drug treatment should be reserved for persistent or severe hypertension with careful monitoring due to the increased risk of adverse pregnancy outcomes [11]. We found that up to 1 % of women under 30 years of age, 1 % to 4 % between 31 and 40 years, and up to 7 % of those aged 44 years were using agents acting on the renin-angiotensin system from risk category D. For pregnant women, antihypertensives with lower risk category might be considered.

Antiepileptic medicines such as valproate [12–14] and carbamazepine [12, 14–16] are associated with an increased risk for major congenital malformations, including neural tube defects and should be prescribed with caution in pregnancy [17]. Topiramate is associated with an increased risk for oral clefts [18, 19] and the Food and Drug Administration (FDA) in US issued a change in label to warn of the potential risk [20]. Lamotrigine might be associated with an increase in the absolute risk for oral clefts [18]. Collectively between 2 % and 4 % of women used anti-epileptics from risk category D in each year of age. Without data on indication, we cannot assess whether there were alternative options. Loss of seizure control during pregnancy is also a risk, and this level of use may represent a consideration of risks and benefits.

The risk of congenital malformations associated with maternal use of antidepressants, especially selective serotonin reuptake inhibitors (SSRIs) as a group, appears to be small and mostly non-significant [21–23], however, meta analyses have found an association between specific SSRIs and birth defects. An increased risk for some heart defects was observed with paroxetine use (Category D) [8, 24, 25] as well as fluoxetine [8] (currently Category C), while the evidence for sertraline (Category C) is conflicting [8, 22, 23]. Overall, sertraline was the most commonly dispensed of these three antidepressants. Fluoxetine had the highest rates of use in women below 20 years, and paroxetine was used by 1 % or less of women in each year of age, despite the availability of antidepressants with less risk potential.

We analyzed data from a national dataset capturing prescription data of all Australians. We included both general and concessional beneficiaries as since 2012 all below co-payment prescriptions are recorded in the PBS data, allowing for generalization of the results to the entire Australian population. Our study was not intended to assess medicine use in pregnancy. Our results are descriptive only addressing national use of prescribed medicines in Australian women of child-bearing age with special focus on medicines that may be teratogenic. We could not capture medicines that are not listed under the PBS and are available over the counter or as private prescriptions.

## Conclusion

It is important for medical practitioners to offer counselling around pregnancy planning and the risk of birth defects when prescribing moderate or high risk teratogens to women of child-bearing age. For the antihypertensives and some anti-epileptics, alternative medicines with lower risk categorization are available.

## Abbreviations

WA: Western Australia
PBS: Pharmaceutical benefits scheme
ATC: Anatomical therapeutic chemical classification
DHS: Department of human services
FDA: Food and drug administration
US: United States
SSRI: Serotonin reuptake inhibitors
ACE inhibitors: Angiotensin converting enzyme inhibitors
ARBs: Angiotensin II receptor antagonists

## References

[1] Hilder L, Zhichao Z, Parker M, Chambers G (2014). Australia'a mothers and babies 2012. Perinatal statistics series no.30. Cat. no. PER 69.

[2] Mitchell A (2003). Systematic identification of drugs that cause birth defects - A new opportunity. New Engl J Med.

[3] Australian Government Department of Health. Therapeutic Goods Administration. Prescribing medicines in pregnancy database. https://www.tga.gov.au/prescribing-medicines-pregnancy-database.

[4] Pearson S, Pesa N, Langton J, Drew A, Faedo M, Robertson J (2015). Studies using Australia's Pharmaceutical Benefits Scheme data for pharmacoepidemiological research: a systematic review of the published literature 1987–2013. Pharmacoepidemiol Drug Saf.

[5] Colvin L, Slack-Smith L, Stanley FJ, Bower C (2009). Pharmacovigilance in pregnancy using population-based linked datasets. Pharmacoepidemiol Drug Saf.

[6] Australian Government. Department of Health. The Schedule of Pharmaceutical Benefits. http://www.pbs.gov.au/pbs/home.

[7] WHO Collaborating Centre for Drug Statistics Methodology (2012). The Anatomical Therapeutic Chemical Classification System.

[8] Reefhuis J, Devine O, Friedman JM, Louik C, Honein MA, National Birth Defects Prevention S (2015). Specific SSRIs and birth defects: bayesian analysis to interpret new data in the context of previous reports. Bmj.

[9] Australian Bureau of Statistics. Australian Demographic Statistics, Sep 2014 (released 26 Mar 2015). http://www.abs.gov.au/AUSSTATS/abs@.nsf/Lookup/3101.0Main+Features1Sep%202014?OpenDocument.

[10] Australian Bureau of Statistics. Births, Australia, 2013 (released 23 Oct 2014) http://www.abs.gov.au/AUSSTATS/abs@.nsf/DetailsPage/3301.02013?OpenDocument.

[11] Donovan P (2012). Hypertensive disorders of pregnancy. Aust Prescr.

[12] Werler MM, Ahrens KA, Bosco JLF, Mitchell AA, Anderka MT, Gilboa SM (2011). Use of Antiepileptic Medications in Pregnancy in Relation to Risks of Birth Defects. Ann Epidemiol.

[13] Jentink J, Loane MA, Dolk H, Barisic I, Garne E, Morris JK (2010). Valproic Acid Monotherapy in Pregnancy and Major Congenital Malformations. New Engl J Med.

[14] Meador K, Reynolds MW, Crean S, Fahrbach K, Probst C (2008). Pregnancy outcomes in women with epilepsy: A systematic review and meta-analysis of published pregnancy registries and cohorts. Epilepsy Res.

[15] Matalon S, Schechtman S, Goldzweig G, Ornoy A (2002). The teratogenic effect of carbamazepine: a meta-analysis of 1255 exposures. Reprod Toxicol.

[16] Jentink J, Dolk H, Loane MA, Morris JK, Wellesley D, Garne E, et al. Intrauterine exposure to carbamazepine and specific congenital malformations: systematic review and case–control study. Brit Med J. 2010;341. 10.1136/bmj.c6581 PMC2996546 21127116

[17] Crawford P (2005). Best practice guidelines for the management of women with epilepsy. Epilepsia.

[18] Holmes LB, Hernandez-Diaz S (2012). Newer anticonvulsants: lamotrigine, topiramate and gabapentin. Birth Defects Res A Clin Mol Teratol.

[19] Mines D, Tennis P, Curkendall SM, Li DK, Peterson C, Andrews EB (2014). Topiramate use in pregnancy and the birth prevalence of oral clefts. Pharmacoepidemiol Drug Saf.

[20] FDA warning and chage of label Topamax (topiramate). Label change - risk for development of cleft lip and/or cleft palate in newborns. Available as generic topiramate. 2011. http://www.fda.gov/Safety/MedWatch/SafetyInformation/SafetyAlertsforHumanMedicalProducts/ucm245777.htm.

[21] Kallen BAJ, Olausson PO (2007). Maternal use of selective serotonin re-uptake inhibitors in early pregnancy and infant congenital malformations. Birth Defects Res A.

[22] Louik C, Lin AE, Werler MM, Hernandez-Diaz S, Mitchell AA (2007). First-trimester use of selective serotonin-reuptake inhibitors and the risk of birth defects. New Engl J Med.

[23] Kornum JB, Nielsen RB, Pedersen L, Mortensen PB, Norgaard M (2010). Use of selective serotonin-reuptake inhibitors during early pregnancy and risk of congenital malformations: updated analysis. Clin Epidemiol.

[24] Bar-Oz B, Einarson T, Einarson A, Boskovic R, O'Brien L, Malm H (2007). Paroxetine and congenital malformations: Meta-analysis and consideration of potential confounding factors. Clin Ther.

[25] Wurst KE, Poole C, Ephross SA, Olshan AF (2010). First Trimester Paroxetine Use and the Prevalence of Congenital, Specifically Cardiac, Defects: A Meta-Analysis of Epidemiological Studies. Birth Defects Res A.

